# Supplementary material for: Origami-based tunable truss structures for non-volatile mechanical memory operation
Source: Nat Commun. 2017 Oct 17;8:962. doi: 10.1038/s41467-017-00670-w (PMC5714951; doi:10.1038/s41467-017-00670-w)
Supplement: Supplementary file 1 — Supplementary Information [file 41467_2017_670_MOESM1_ESM.pdf]

Description of Supplementary Files

File Name: Supplementary Information
Description: Supplementary Figures, Supplementary Notes, and Supplementary References.

File Name: Supplementary Movie 1
Description: Comparison between paper-based and truss-based TCO models. We use a bistable configuration for both models, and we evidently observe the warping of facets from the paper model.

File Name: Supplementary Movie 2
Description: Compression test on a TCO-based truss structure in a single-cell level. The configuration used is bistable.

File Name: Supplementary Movie 3
Description: Distinctive behavior of four TCO-based truss structures presented in this study: monostable model; bistable model; zero-stiffness model; and bifurcation model.

File Name: Supplementary Movie 4
Description: Bifurcation test on a TCO-based truss structure in a single-cell level. The left panel in the movie shows an unstable branch of folding by using mechanical constraints. The right panel shows a stable branch without any constraints.

File Name: Supplementary Movie 5
Description: Single-bit operation of the TCO-based truss structure composed of a pair of monostable cells.

File Name: Supplementary Movie 6
Description: Two-bit memory operation of the TCO-based truss structure composed of two pair of monostable cells. This process shows the transition from `00' to ` 11'.

File Name: Supplementary Movie 7
Description: Two-bit memory operation of the TCO-based truss structure composed of two pair of monostable cells. This process shows the transition from `01' to `10'.

File Name: Peer Review File

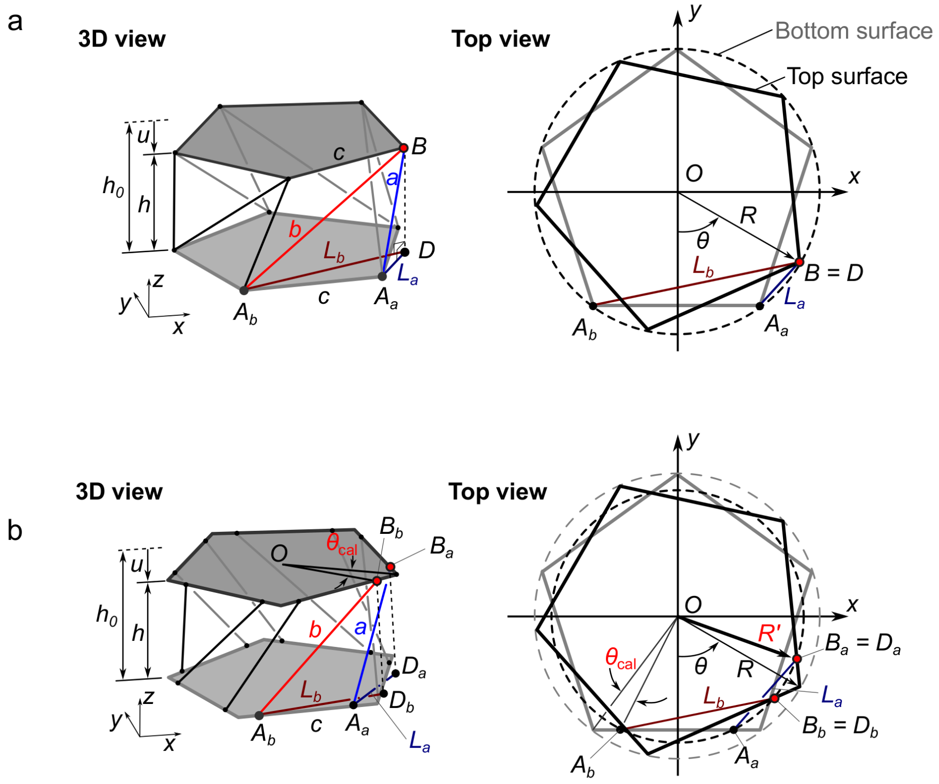

Supplementary Figure 1: **Original and modified TCO model.** (**a**) 3D (left) and top (right) views of the original TCO unit cell are shown. (**b**) We modify the geometry of the TCO by separating a single vertex (e.g., B in (**a**)) into two vertices (e.g., B_a and B_b in (**b**)). The differences between the original TCO model and modified TCO model are characterized by θ_{cal} and R'.

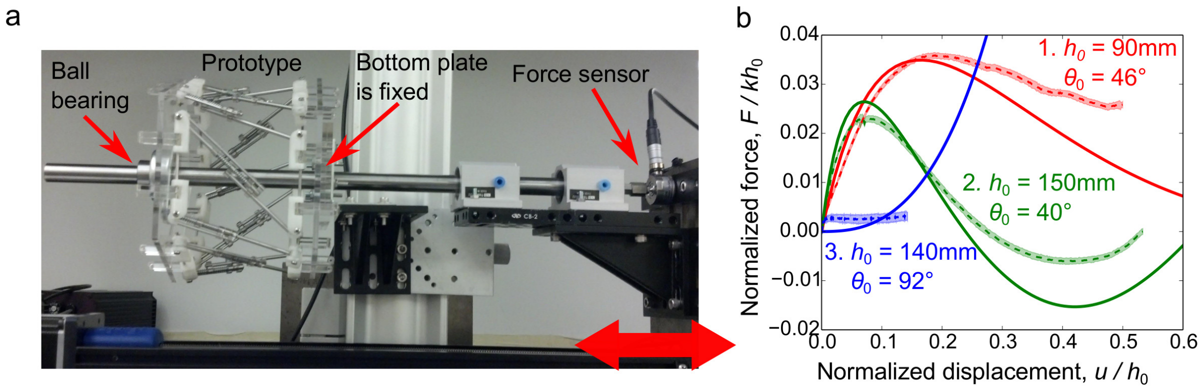

Supplementary Figure 2: **Compression test setup and experimental/analytical data.** (**a**) The photograph of the compression test setup is shown. The fabricated prototype of the TCO-based truss structure is placed horizontally. The right end of the prototype is fixed, while its left end is attached to a stainless steel shaft via a ball bearing. This allows the left plate to rotate freely with minimal friction. Axial force is measured by a force sensor attached the shaft. (**b**) Dashed curves show measurements of the axial force (normalized by kh_0) as a function of axial compression (normalized by h_0) for three different prototypes, which represent monostable, bistable, and zero-stiffness configurations. Shaded areas represent standard deviations of the measured data. Solid curves show predictions from the potential energy analysis.

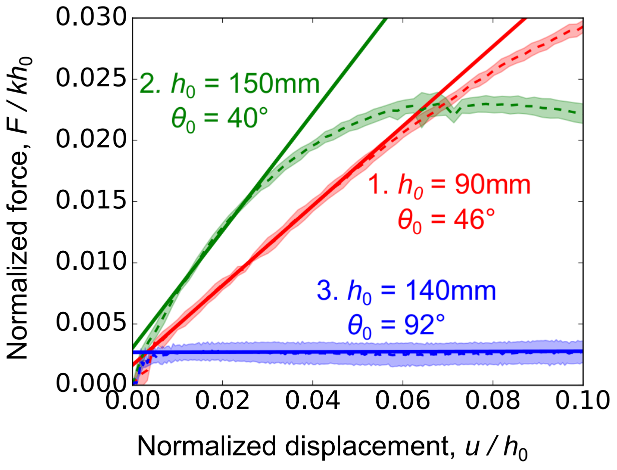

Supplementary Figure 3: **Force-displacement curves for the comparison of stiffness.** We zoom up the force-displacement measurements from the three prototypes near $u/h_0 = 0$. The solid lines are based on the linear approximation of the experimental data in the range between $u/h_0 = 0.01$ and 0.03. It is evident that the zero-stiffness case exhibits a significantly smaller slope (i.e., stiffness) compared to the other cases.

a

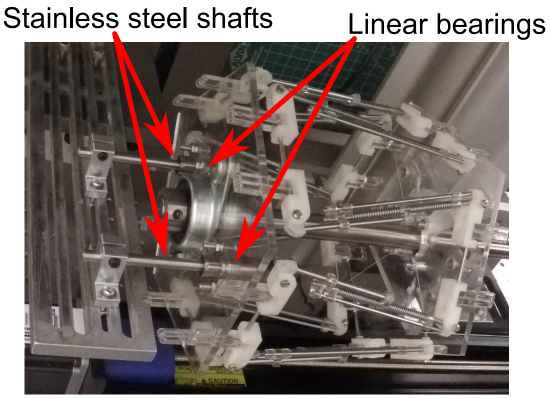

b

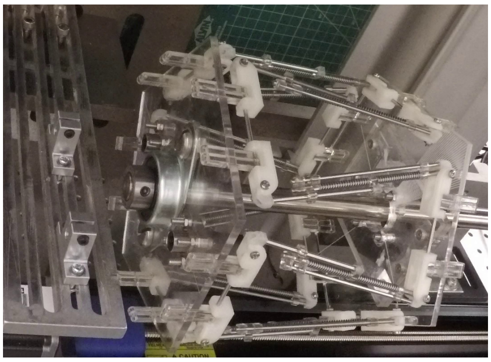

c

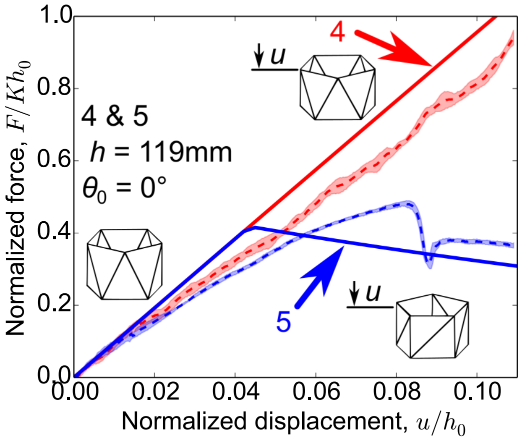

Supplementary Figure 4: **Bifurcation test setup and experimental/analytical data.** (**a**) The photograph shows the compression test setup with constraints on the rotational motion of the left plate of the prototype. Two stainless steel shafts and linear bearings are used to allow the translational motion without triggering the rotational motion. (**b**) The test setup without constraints on the rotational motion is shown. (**c**) Force-displacement measurements for the two cases show a bifurcation behavior. Here, dashed and solid curves indicate the measured experimental results and the predictions from the energy analysis, respectively. The arrows 4 and 5 indicate the unstable and stable cases respectively.

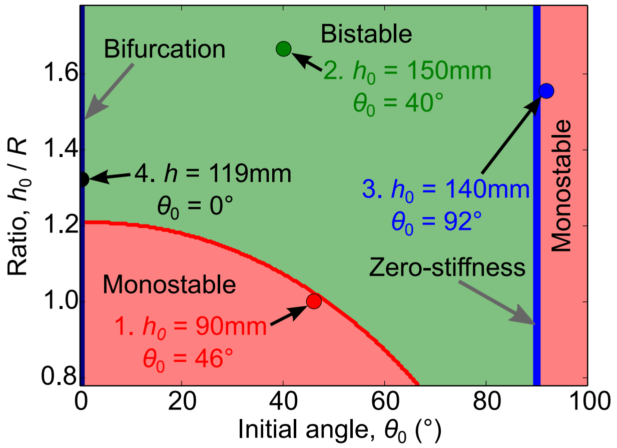

Supplementary Figure 5: **Tunable behavior of the TCO.** Tunable behavior of the TCO-based unit cell is examined numerically in various combinations of initial configurations (i.e., h_0 and θ_0). We use θ_{cal} = 9.7° for all calculations. Red and green regions indicate monostable and bistable behaviors respectively. If $\theta_0 = 90°$ (blue solid line), the unit cell shows the zero-stiffness mode. The black solid line ($\theta_0 = 0°$) indicates that the system shows the bifurcation behavior in the investigation range of the height ratio.

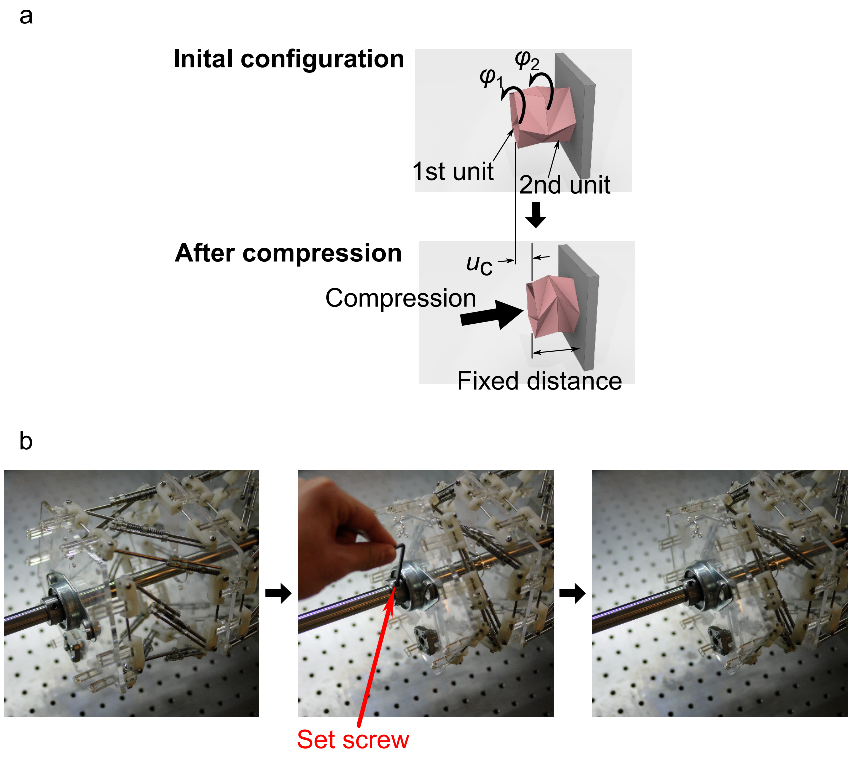

Supplementary Figure 6: **TCO-based mechanical memory storage.** (**a**) Two monostable TCO-based unit cells ($h_0 = 90$ mm, $\theta_0 = \pm 46°$) are connected horizontally as shown in the schematic illustration. The upper panel illustrates the initial configurations under no external force and torque. We apply pre-compression to this system (denoted by u_C), and we fix the distance between leftmost and rightmost plates as shown in the lower panel. The rotation of the leftmost surface of the system is φ_1 measured with respect to its initial configuration. (**b**) Photographs show the preparation of the system with pre-compression by tightening a set screw of the bearing which is attached to the cross-section of the TCO-based unit cell.

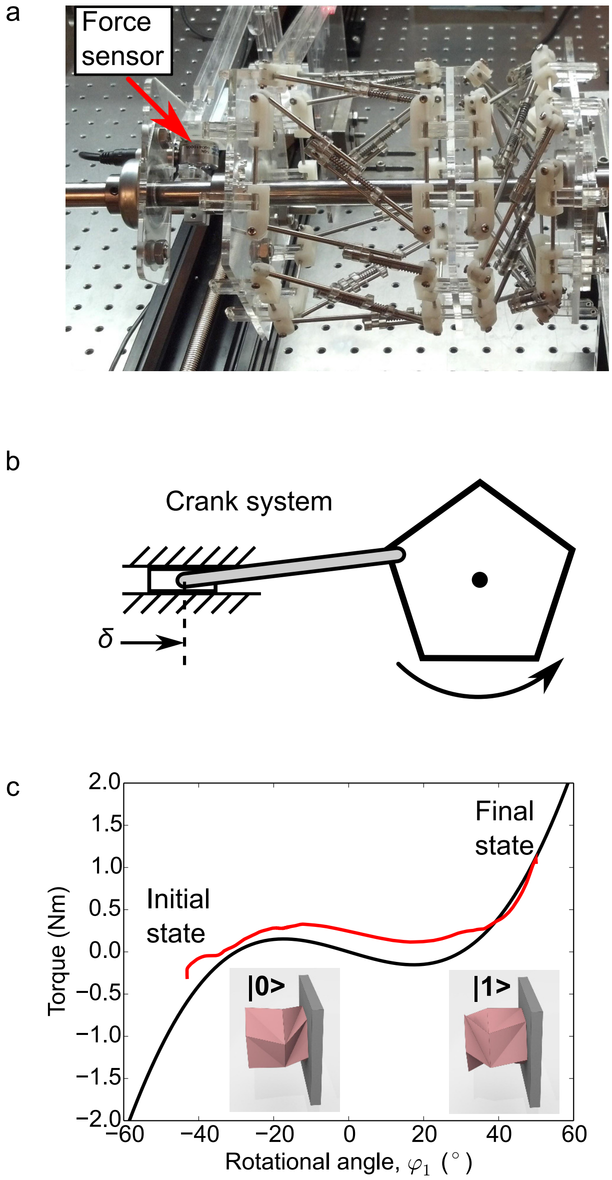

Supplementary Figure 7: **Single bit operation for memory storage.** (**a**) Photograph shows the test setup for measuring the relationship between torque and φ_1. We obtain the torque information by using the force sensor attached to the crank system, which is operated by the linear stage. (**b**) The crank system is used to convert the translational motion (δ) into the rotational motion. We measure/control δ to impose an accurate amount of the rotation of the system. (**c**) Measurements of the torque as a function of the rotational angle (red curve) show a good agreement with the prediction from the energy analysis (black curve). The insets show the graphical illustrations of the ‘0’ and ‘1’ states.

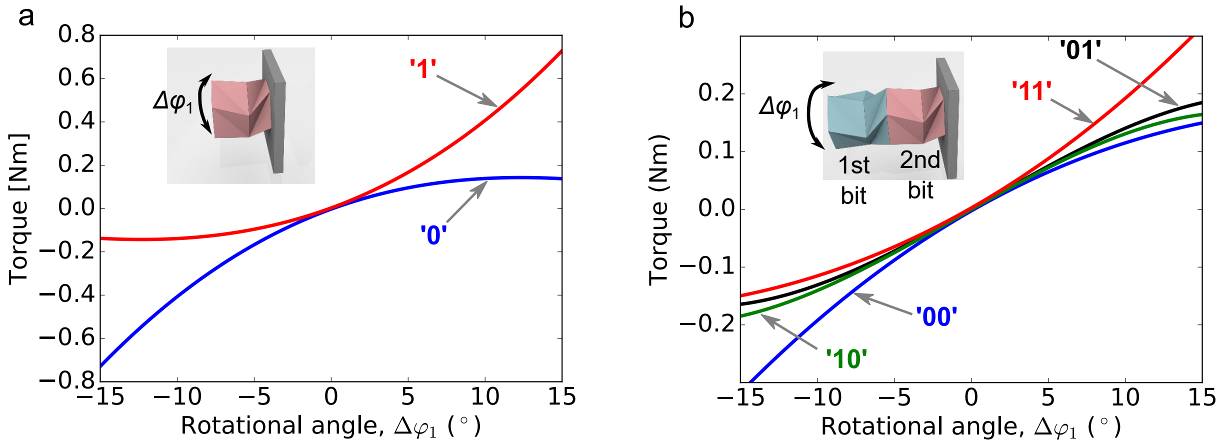

Supplementary Figure 8: **Readout of the bit information for the single-bit and the two-bit systems.** (**a**) We consider the single-bit system with (h_0, θ_0) = (90 mm, $\pm 46°$), and we apply angular perturbations ($\Delta\varphi_1$) around each local minimum energy state ($\varphi_1 = -29°$ for '0' and $\varphi_1 = +29°$ for '1'). The blue and red curves show torque change around '0' and '1' respectively. (**b**) The same perturbation of the rational angle is applied to the two-bit system with (h_0, θ_0) = (90 mm, [$+46°$, $-46°$, $+46°$, $-46°$]), and we obtain torque change. The blue, green, black, and red curves indicate torque change around '00', '10', '01', and '11' respectively.

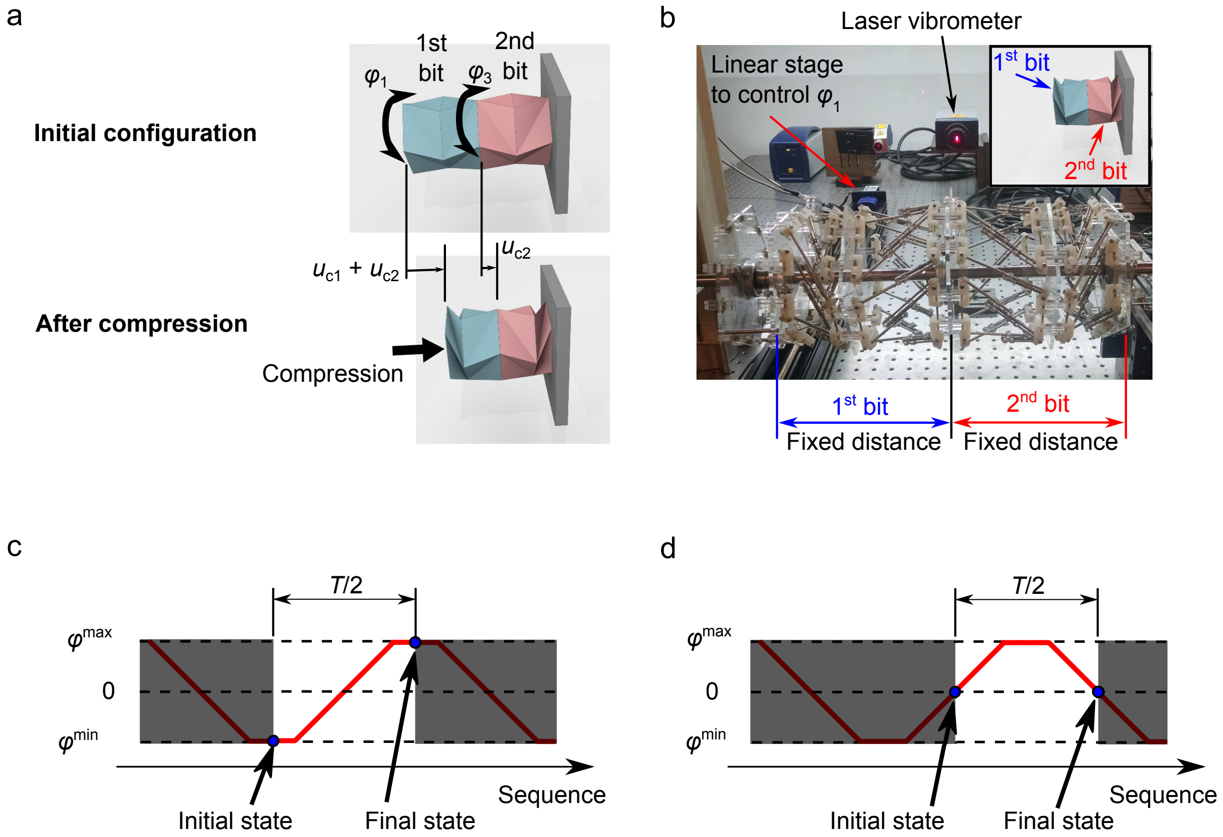

Supplementary Figure 9: **Configuration of TCO cells for two-bit memory operation.** (**a**) Four TCO-based unit cells are used to form a two-bit memory as shown in the schematic illustration. To achieve four different states, we apply compression to the first and second bits, which is denoted by u_{C1} and u_{C2}. (**b**) Photograph shows the test setup for the two-bit memory operation. φ_1 is controlled by the motor-driven linear stage, and φ_3 is measured by the laser doppler vibrometer. (**c**–**d**) Pulse input is designed based on a trapezoid-shaped waveform, whose amplitude changes between maximum and minimum values of φ. The shape of the pulse input is determined depending on the initial state and duration.

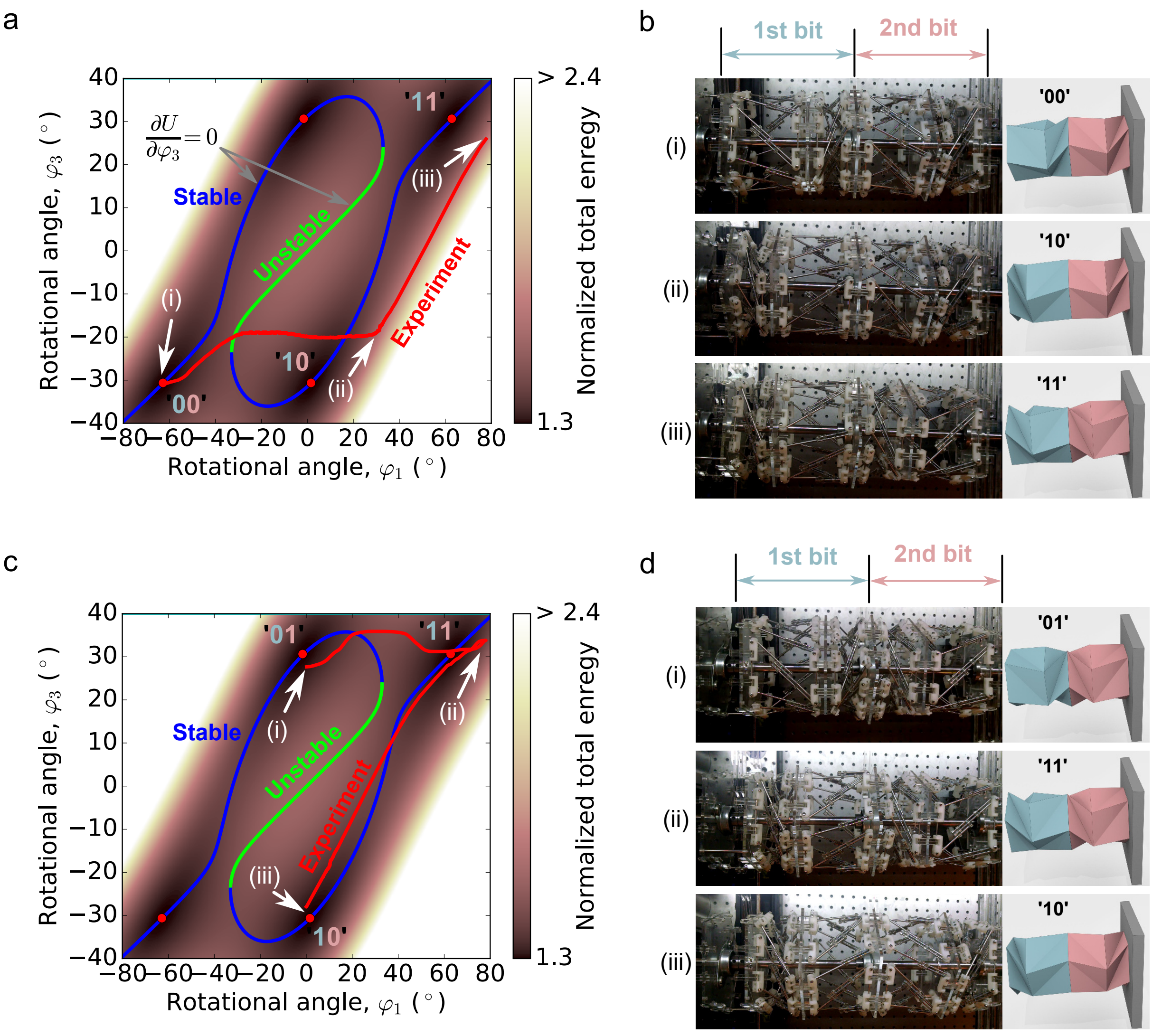

Supplementary Figure 10: **Operation of the two-bit memory.** (**a**) The normalized energy of the two-bit system as a function of φ_1 and φ_3 is shown. The blue and green curves are obtained from $\partial U / \partial \varphi_3 = 0$, and blue and green lines indicate stable and unstable paths, respectively. The experimental result for the trajectory of the two-bit memory operation is shown in a red curve. Red circles are the states where the system takes local minimum energy. The experiment starts from (i) and changes the state to (ii) and eventually to (iii). (**b**) Photographs of the prototype show the transition from '00' to '10', then to '11'. (**c**) Trajectory for the case of '01' $\rightarrow$ '10' is shown in a red curve. (**d**) Photographs of the prototype shows the transition from '01' to '11', then to '10'.

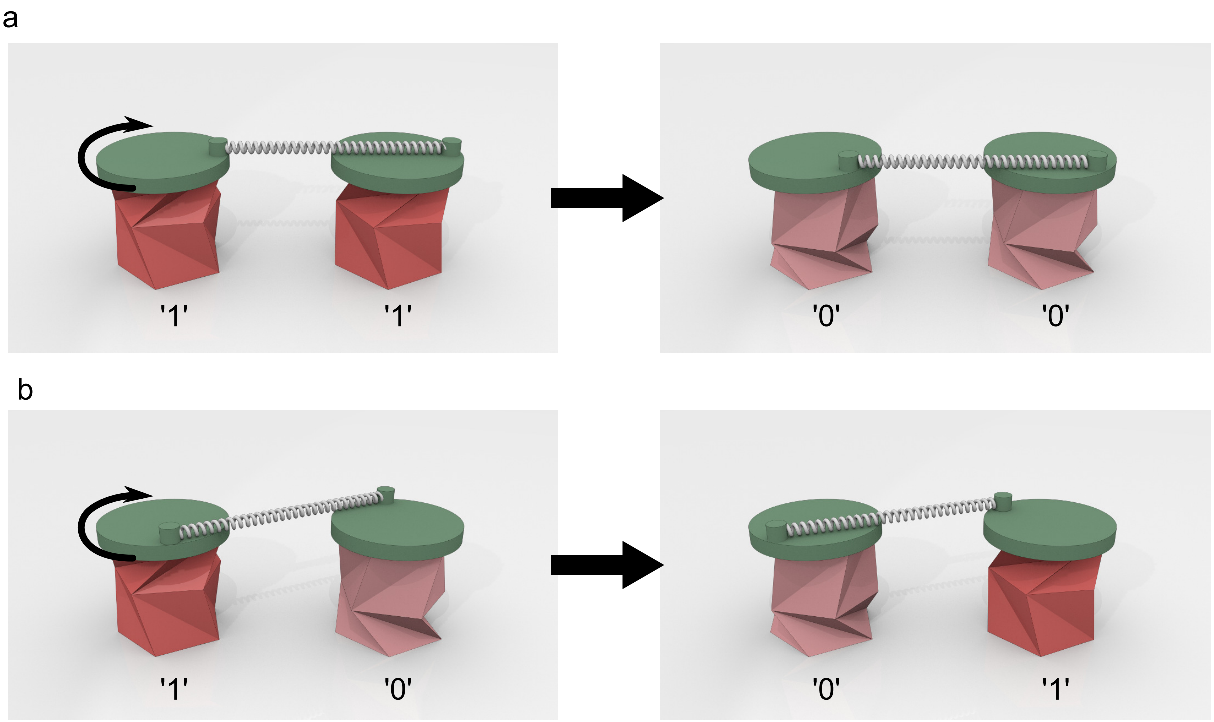

Supplementary Figure 11: **Conceptual illustration of extended TCO-based mechanical memory in horizontal connections.** (**a**) The top disks of two neighboring bits are connected by a linear spring. If we apply clock-wise torque to the left bit, the right bit transforms from ‘1’ $\rightarrow$ ‘0’. (**b**) If we connect two adjacent bits in a diagonal direction and apply the same torque to the left bit, the right bit transforms from ‘0’ $\rightarrow$ ‘1’.

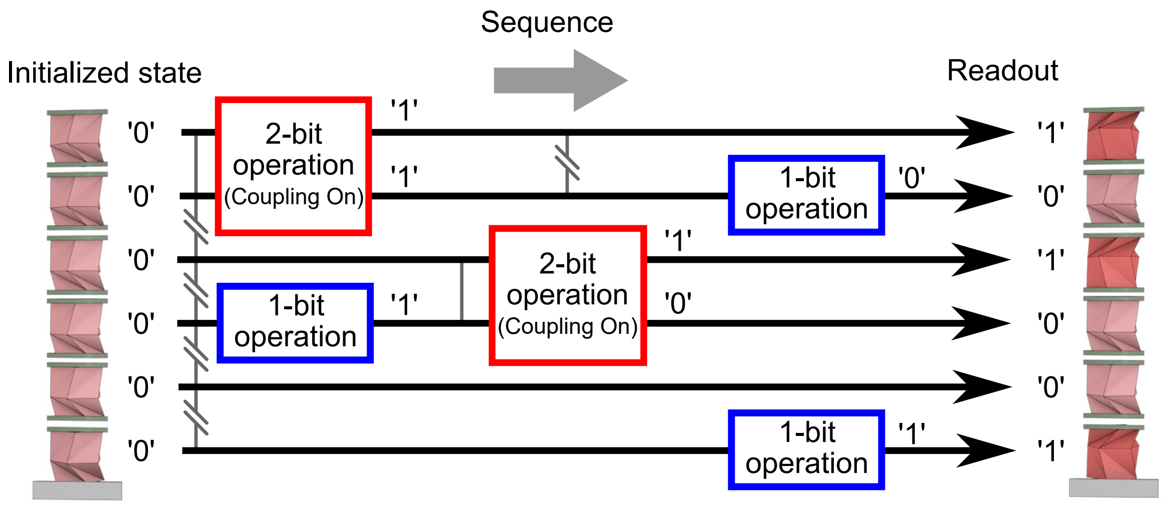

Supplementary Figure 12: **Conceptual illustration of TCO-based mechanical computing.**

Schematic illustration of calculation flow is shown.

Supplementary Note 1: Modeling of the TCO-based truss structure

In this study, we approximate the TCO cell into a truss-like structure composed of interfacial polygons and axial members (Supplementary Fig. **1**a). This is to eliminate the effect of surface warping in the TCO cells, while preserving their key characteristics, such as the coupled dynamics of the axial and torsional motions (Supplementary Movie 1). We model the folding behavior of the TCO-based truss structure using the principle of minimum potential energy [1]. Here we briefly describe this modeling process. First, we derive the expressions of the length change of crease lines a and b, as the TCO cell experiences axial and angular deformations. Let h be the height of the unit cell and R be a radius of the circle circumscribing the cross-section. Then, we obtain the distance between vertex A_a (A_b) and D as follows:

$$\begin{aligned} L_a &= 2R\sin\left(\frac{\theta}{2} - \frac{\pi}{2n}\right) \\ L_b &= 2R\sin\left(\frac{\theta}{2} + \frac{\pi}{2n}\right) \end{aligned} \tag{S1}$$

where θ is a relative angle of rotation, which is defined as an angle between OB and the perpendicular bisector of A_aA_b (see Supplementary Fig. **1**a).

Also, from the geometry of the TCO, we obtain

$$\begin{aligned} L_a^2 + h^2 &= a^2 \\ L_b^2 + h^2 &= b^2, \end{aligned} \tag{S2}$$

Then, substituting Eq. (S1) into Eq. (S2), we can calculate the length of each crease lines as follows:

$$\begin{aligned} a &= \sqrt{(h_0 - u)^2 + 4R^2\sin^2\left(\frac{\varphi}{2} + \frac{\theta_0}{2} - \frac{\pi}{2n}\right)}, \\ b &= \sqrt{(h_0 - u)^2 + 4R^2\sin^2\left(\frac{\varphi}{2} + \frac{\theta_0}{2} + \frac{\pi}{2n}\right)}. \end{aligned} \tag{S3}$$

Here h_0 and θ_0 are the initial height and relative angle, respectively, and u and φ are the axial and rotational displacements, which are defined as $u = -(h - h_0)$ and $\varphi = \theta - \theta_0$. It is noted that the maximum limit of the rotational angle (φ_{max}) is determined by

$$\varphi_{max} = \left(\pi - \frac{\pi}{n}\right) - \theta_0. \tag{S4}$$

If $\varphi = \varphi_{max}$, the crease lines intersect each other, limiting the maximum axial and angular deformation of the TCO cell.

Based on Eq. (S3), the total elastic energy is calculated as

$$U = \frac{1}{2}nk(a - a_0)^2 + \frac{1}{2}nk(b - b_0)^2, \tag{S5}$$

where a_0 and b_0 are initial length of a and b, and k is a spring constant of the truss members. Here we assume that all truss members exhibit identical axial stiffness. Also, the work done on this system is

$$W = Fu + T\varphi, \tag{S6}$$

where F and T are the external force and torque respectively. Then, by using the expressions of the elastic energy and work in Eq. (S5) and Eq. (S6), the total potential energy (Π) can be expressed as [1]:

$$\Pi(u, \varphi) = U - W = \frac{1}{2}nk(a - a_0)^2 + \frac{1}{2}nk(b - b_0)^2 - Fu - T\varphi. \tag{S7}$$

We apply the principle of minimum total potential energy (i.e., $\delta\Pi = 0$) [1] to Eq. (S7) as follows:

$$\partial\Pi/\partial u = 0 \quad \text{and} \quad \partial\Pi/\partial\varphi = 0. \tag{S8}$$

As a result, we obtain expressions of F and T in terms of u and φ as follows:

$$\begin{aligned} F(u, \varphi) &= nk\,(u - h_0)\left(2 - \frac{a_0}{a} - \frac{b_0}{b}\right), \\ T(u, \varphi) &= nkR^2\left\{\left(1 - \frac{a_0}{a}\right)\sin\left(\varphi + \theta_0 - \frac{\pi}{n}\right) + \left(1 - \frac{b_0}{b}\right)\sin\left(\varphi + \theta_0 + \frac{\pi}{n}\right)\right\}. \end{aligned} \tag{S9}$$

Supplementary Note 2: Modified TCO model

In the original TCO model, axial truss members a and b intersect at vertices that are positioned on the surface of interfacial polygons (see Supplementary Fig. **1**a). Such intersecting joints are not easy to fabricate, when we build a prototype with mechanical joints. Thus, to secure enough space for mechanical joints that connect axial members to the interfacial polygons, we modify the original TCO model. Specifically, we separate a single vertex (e.g., B in

Supplementary Fig. **1**a) into a pair of vertices (e.g., B_a and B_b in Supplementary Fig. **1**b). The difference between the original model and the modified model can be characterized by a shifted angle of the vertices (θ_{cal} in the 3D view of Supplementary Fig. **1**b) and a modified radius of these vertices (R' in the top view of Supplementary Fig. **1**b).

We can reflect these modifications in Eq. (S3) as follows:

$$\begin{aligned} a &= \sqrt{(h_0-u)^2 + 4R'^2 \sin^2\left(\frac{\varphi}{2}+\frac{\theta_0}{2}-\frac{\pi}{2n}+\theta_{\text{cal}}\right)} \\ b &= \sqrt{(h_0-u)^2 + 4R'^2 \sin^2\left(\frac{\varphi}{2}+\frac{\theta_0}{2}+\frac{\pi}{2n}-\theta_{\text{cal}}\right)} \end{aligned} \tag{S10}$$

where

$$R' = \frac{R\cos\left(\frac{\pi}{n}\right)}{\cos\left(\frac{\pi}{n}-\theta_{\text{cal}}\right)}. \tag{S11}$$

Then, Eq. (S9) also needs to be modified accordingly as follows:

$$\begin{aligned} F(u,\varphi) &= nk\,(u-h_0)\left(2-\frac{a_0}{a}-\frac{b_0}{b}\right) \\ T(u,\varphi) &= nk(R')^2\left\{\left(1-\frac{a_0}{a}\right)\sin\left(\varphi+\theta_0-\frac{\pi}{n}+2\theta_{\text{cal}}\right)+\left(1-\frac{b_0}{b}\right)\sin\left(\varphi+\theta_0+\frac{\pi}{n}-2\theta_{\text{cal}}\right)\right\}. \end{aligned} \tag{S12}$$

Note that a and b in Eq. (S12) are based on the expressions in Eq. (S10), which include the modification parameters θ_{cal} and R'. In this manuscript, the computation results of the TCO's folding behavior are based on this modified model.

Supplementary Note 3: Compression test setup and measurement data

In this study, we conduct a series of compression tests on the fabricated TCO prototypes. Supplementary Fig. **2**a shows a unit-cell prototype placed horizontally on the load frame (see also Supplementary Movie 2). In this setup, the right end of the prototype is fixed in both translational and rotational directions. The polygon on the left side is supported by a stainless steel shaft. We use a ball bearing to grab the shaft at the tip, so that the left polygon can rotate freely with minimal friction. The other end of the shaft is connected to the motor-driven linear

stage (BiSlider, Velmex). We control the axial compression of the prototype by translating the shaft along the horizontal direction (see the arrow in Supplementary Fig. **2**a). Note that by using this shaft system, we apply a desired compression to the system, while allowing free rotational motions and restricting any bending motions of the prototype. The axial force transmitted through the shaft is measured by a force sensor located between the right tip of the shaft and the linear stage (LUX-B-50N-ID, Kyowa).

By using the compression test setup, we obtain force-displacement data. Supplementary Fig. **2**b shows the measured force-displacement curves (dashed curves) for (h_0, θ_0) = (90 mm, 46°), (150 mm, 40°), and (140 mm, 92°). The corresponding analytical results are also shown in solid curves. As noted in the main text, these curves show remarkably different behaviors, depending on the choice of the TCO's geometry in terms of h_0 and θ_0. The monostable model ($h_0 = 90$ mm, $\theta_0 = 46°$) shows a maximum peak around u/kh_0 of 0.16, and after this maximum peak, the slope of the force-displacement curve becomes negative, i.e., negative stiffness (red curves in Supplementary Fig. **2**b). The bistable model ($h_0 = 150$ mm, $\theta_0 = 40°$) indicates the transition from compressive force to tensile force during folding (green curves in Supplementary Fig. **2**b).

Lastly, for the zero-stiffness model, the measurement indicates that the slope of the force-displacement is nearly zero at the initial stage (blue curves in Supplementary Fig. **2**b). This zero-stiffness effect is more evident if we zoom up the region near $u/h_0 = 0$ (Supplementary Fig. **3**). By applying the linear approximation to the measurements between u/h_0 = 0.01 and 0.03, we can compare the slope of the force-displacement curves among the different prototypes (see the solid lines in Supplementary Fig. **3** for the linearization). The reason that we apply the linearization not from $u/h_0 = 0$ is due to the static friction in the mechanical joints (see the initial force required to overcome this friction in all curves in Supplementary Fig. **3**). The slope of these non-dimensionalized graphs are 0.326, 0.479, and 0.000861 for monostable, bistable, and zero-stiffness cases, respectively. As noted in the main manuscript, the slope of

this zero-stiffness case corresponds to mere 0.26% and 0.18% compared to the monostable and bistable cases. This successfully verifies the feasibility of zero-stiffness structure using this TCO architecture, making it potentially applicable for impact mitigation purposes.

Overall, the force-displacement curves obtained from the compression tests are in qualitative agreement with the analytical predictions (Supplementary Fig. **2**b). The discrepancy between the experimental and analytical data may be attributed to the friction in the mechanical joints of the prototypes. In fact, the application of grease to these joints significantly improved the quality of the experimental data, particularly in the earlier part of the compression tests. In the main manuscript, we numerically integrated these force-displacement curves and presented the total energy profiles (Fig. 2 in the main text). This was to highlight tailorable monostable/bistable features (e.g., double-well potential) in the TCO systems.

Supplementary Note 4: Zero-stiffness mode

Here we mathematically derive how to meet the zero-stiffness condition using the truss-based TCO architecture. We first linearize Eq. (S3) around the undeformed, initial state ($u = \varphi = 0$):

$$\begin{aligned} a &\approx a_0 + \alpha_u u + \alpha_\varphi \varphi \\ b &\approx b_0 + \beta_u u + \beta_\varphi \varphi, \end{aligned} \tag{S13}$$

where a_0 and b_0 are initial length of a and b respectively, and

$$\begin{aligned} \alpha_u &= -\frac{h_0}{a_0} \\ \alpha_\varphi &= \frac{R^2 \sin(\theta_0 - \pi/n\ )}{a_0} \\ \beta_u &= -\frac{h_0}{b_0} \\ \beta_\varphi &= \frac{R^2 \sin(\theta_0 + \pi/n\ )}{b_0}. \end{aligned} \tag{S14}$$

After the linearization, Eq. (S13) can be expressed in a matrix form as

$$\boldsymbol{e} \approx \boldsymbol{A}\boldsymbol{\xi} \tag{S15}$$

where

$$\boldsymbol{A} = \begin{bmatrix} \alpha_u & \alpha_\varphi \\ \beta_u & \beta_\varphi \end{bmatrix},$$

$$\boldsymbol{e} = \begin{bmatrix} a - a_0 \\ b - b_0 \end{bmatrix},$$

$$\boldsymbol{\xi} = \begin{bmatrix} u \\ \varphi \end{bmatrix}.$$

Here, $\boldsymbol{e}$ represents the length change of the crease lines, a and b. To fulfill the zero-stiffness condition, the length change of the crease lines (i.e., $\boldsymbol{e}$) need to be infinitesimal given external excitations (i.e., $\boldsymbol{\xi}$). Mathematically, this implies $\det \mathbf{A} = \alpha_u\beta_\varphi - \alpha_\varphi\beta_u = 0$. By using the definitions of the coefficients in Eq. (S14), we obtain

$$\sin\left(\frac{\pi}{n}\right)\cos\left(\theta_0\right) = 0 \tag{S16}$$

This means, if the initial angle (θ_0) is $\pi/2$, $\det \mathbf{A}$ becomes zero, which leads to the zero-stiffness mode. Note that this condition can be also met by having $n \to \infty$. Thus, a TCO cell with a large number of truss members that connect the top and bottom polygons (i.e., polygons with numerous vertices) will also exhibit a small stiffness under compression. It should be noted that Eq. (S16) contains only θ_0 and n. Therefore, the zero-stiffness mode is not affected by the spring constant of the truss members (k) or the initial height of the TCO cell (h_0).

Supplementary Note 5: Bifurcation test setup and measurement data

In this study, we also conduct the compression test on the bifurcation model ($h_0 = 119$ mm, $\theta_0 = 0°$). As shown in Fig. 2d in the main text, this bifurcation model has two distinctive modes of folding: an unstable mode that undergoes only axial motions without rotation, and a stable mode that develops combined axial and rotational motions. To verify this bifurcation behavior, we consider two experimental setups. The first one is with the constraints on the rotational

motion of the prototype (Supplementary Fig. **4**a), and the other one is without any constraints (Supplementary Fig. **4**b). For application of the constraints on the rotational motion of the prototype, we use a pair of stainless steel shafts and linear bearings, which allow the prototype to translate in the axial direction without any rotational motions (Supplementary Fig. **4**a). The setup without any constraints is identical to the test configuration described in Supplementary Note 3. See Supplementary Movie 4 for the comparison of these two operations.

Supplementary Fig. **4**c shows the experimental results (dashed curves) and analytical predictions (solid curves). In the unstable case (red curves indicated by arrow 4), the axial force increases monotonically from u/h_0 = 0 to 0.1. As shown in the inset, the TCO cell compresses without twisting motions. This unstable mode is possible only if there exist constraints that prevent any onset of rotational motions. On the other hand, if the constrains are removed, the measurement shows a kink point where the slope of the force-displacement curve changes to negative (blue curves indicated by arrow 5 in Supplementary Fig. **4**c). Note that the TCO cell experiences a twisting motion in one or the other direction after the bifurcation point, as illustrated by the inset. We observe noticeable discrepancies between the experimental and analytical results, particularly in terms of the bifurcation point. Again, this is probably due to the friction existing in the prototypical systems.

Supplementary Note 6: Tunable stability of the TCO-based truss structure

In this study, we observe versatile nature of the TCO-based truss structure as we alter its geometry. Particularly, the stability of the system can be manipulated by changing its two geometrical parameters: initial twist angle (θ_0) and height (h_0). To examine such tunable stability of the TCO-based unit cell, we numerically analyze its stability for various initial configurations. The process is as follows. We first calculate the elastic energy (U) as a function of axial compression (u) by following the trajectory of the minimum energy valley (e.g., see Fig. 2 in the main manuscript). Then, the slope of this energy curve is numerically obtained by using

$(U_{i+1} - U_i)/(u_{i+1} - u_i)$, where i represents a step for numerical calculations. If the TCO-based structure exhibits the bistable behavior, there exists a transition from a positive to negative slope. Thus, we can judge whether the unit cell shows monostable or bistable behavior.

The results are shown in Supplementary Fig. **5**. We evidently observe monostable and bistable regions. We also mark lines that represent zero-stiffness ($\theta_0 = 90°$) and bifurcation ($\theta_0 = 0°$) modes in the investigation range. Note that the zero-stiffness mode is located at the boundary between the monostable and bistable regions. In the bistable regions, there exist two stable states, and these two stable states merge at this boundary as the initial angle (θ_0) increases. The four prototypes that we fabricate are also marked as circles in the figure.

Supplementary Note 7: Mechanical bit operation for memory storage

To verify our concept of a mechanical bit operation based on the double-well potential, we assemble a pair of monostable TCO cells ($R = 90$ mm, $h_0 = 90$ mm, $\theta_0 = \pm 46°$) that are connected horizontally by sharing the middle surface. As shown in Supplementary Fig. **6**a, we apply pre-compression (u_C) and fix the distance between the leftmost and rightmost polygons of the structure by using a stainless steel shaft and a ball bearing. Similar to the single cell test described in Supplementary Note 3, this shaft setup allows a free rotation of the leftmost polygon, while restricting its translational motion as shown in Supplementary Fig. **6**b.

For the operation of the single bit, we control the rotation of the leftmost polygon (denoted by φ_1) by applying torque to the polygon (Supplementary Fig. **7**a). For the accurate control of the imposed rotational angle, we use a crank system attached to the leftmost polygon of the prototype (Supplementary Fig. **7**b). We measure the exact amount of the translational motion (δ) by using a non-contact laser Doppler vibrometer (OFV-505, Polytec), and covert the measurement values to the rotational motion (φ_1). A force sensor (LUX-B-50N-ID, Kyowa) is also attached at the end of the crank arm to calculate the torque applied.

Supplementary Fig. **7**c shows the experimental measurements (red curve) of the torque and

the rotational angle, in comparison to the analytical results (black curve) predicted by the energy analysis. We observe that as we rotate the leftmost polygon (i.e., φ_1 increases), the system changes its state from '0' (i.e., the initial configuration that the right cell is folded) to '1' (i.e., the final configuration that the left cell is folded). This experimental trend agrees well with the prediction from the energy analysis. See Supplementary Movie 5 for the manual bit operation.

Supplementary Note 8: Readout stored bit information

In this study, we have used a laser vibrometer to determine the bit states by measuring the rotational angles φ_1 and φ_3 individually. However, it would be useful to determine the state of both bits only by measuring the response of the first bit *mechanically*. To readout both bits simultaneously, we propose to apply angular perturbations (φ_1) to the origami system on its actuation side (i.e., the left-most cross-section as shown in the insets of Supplementary Fig. **8**) and measure the torque profile. In the case of a single-bit system (Supplementary Fig. **8**a), we consider the initial condition of (h_0, θ_0) = (90 mm, $\pm 46°$) and the pre-compression of u_C = 45 mm (same as the condition described in the main manuscript). In this configuration, there are two local minimum energy states ($\varphi_1 = -29°$ for '0' and $\varphi_1 = +29°$ for '1'). Then, we apply angle perturbation of $\Delta\varphi_1 = \pm 15°$ and measure the torque profile from the left-most side (i.e., the torque profile is measured in the range of $\varphi_1 = -29° \pm 15°$ for '0' case, and $\varphi_1 = +29° \pm 15°$ for '1'). Please note that $\Delta\varphi_1 = \pm 15°$ is chosen not to overcome the energy barrier of the single-bit system, so that we avoid the destruction of the bit information in this perturbation range. Supplementary Fig. **8**a shows the numerical results of the torque profiles for the single-bit case. We obtain two distinctive torque curves, which indicate that the bit information can be extracted by the application of angular perturbations ($\Delta\varphi_1$) and the subsequent reading of the torque profiles.

Similarly, we can extend this readout method to the two-bit case. Here, we numerically consider the case of (h_0, θ_0) = (90 mm, [$+46°$, $-46°$, $+46°$, $-46°$]) and the pre-compression of

u_C = 50 mm and 47.5 mm for the first and second bit, respectively (again, the condition identical to that described in the main manuscript). We first apply rotational perturbations of $\Delta\varphi_1$ = $\pm15°$ at four local minimum energy states: (φ_1, φ_3) = ($-63°$, $-31°$) for '00', ($+2°$, $-31°$) for '10', ($-2°$, $+31°$) for '01', and ($+63°$, $+31°$) for '11'. Supplementary Fig. **8**b shows our calculation results, from which we observe four different curves for each bit state. This again confirms that we can extract the information about the bit state by applying $\Delta\varphi_1$ and measure the corresponding torque. We want to point out that because '01' and '10' cases show similar torque curves, the practical challenge would be a precise measurement of the torque under external influence such as noise and friction. However, we expect that these torque profiles can be further separated by changing u_C values or the initial twist angles (θ_0) imposed on both cells. Also, the dynamic response of the system (e.g., frequency response) can be also measured to predict the stiffness of the system and thus, to read its memory state.

Supplementary Note 9: Two-bit memory operation

By using a pair of single bits (R = 90 mm, h_0 = 90 mm, θ_0 = $\pm46°$), we design a two bit system (Supplementary Fig. **9**a). We apply pre-compressions to the first and second bit, which are denoted by u_{C1} and u_{C2} respectively. Depending on the deformed states of these two bits, the two-bit system exhibits four different states: '00', '01', '10', and '11', where the first and second numbers indicate the first bit (the left pair in blue color in Supplementary Fig. **9**a) and second bit (the right pair in red color), respectively. The state of the system is determined by the overall rotational angles of these two TCO pairs, which are represented by the twisted angles of the first and third polygons, φ_1 and φ_3. In the experiments, we use the crank system to control φ_1 via the motor-driven linear stage, and φ_3 is obtained from the non-contact laser Doppler vibrometer (OFV-505, Polytec). See Supplementary Fig. **9**b for the digital image of the experimental setup.

For the two-bit memory operation, we use a pulse input based on a trapezoid-shaped wave-

form to change the amplitude of φ_1 between maximum and minimum values in a systematic manner. This technique based on trapezoidal input has been used in [4] for the verification of the controlled NOT gate, which also employs two bit units. In this study, we use two different waveforms of the trapezoidal pulse as shown in Supplementary Fig. **9**c and d. For the monotonous increase of the input pulse, we use the waveform in Fig. **9**c, while for the increasing – then decreasing – trend of the input pulse, we employ the waveform in Fig. **9**d. Note that the two pulse inputs are taken from the identical trapezoidal waveform, and the shape of the pulse input is determined by the location of the wave profile selected from the trapezoidal waveform with a half period ($T/2$).

Supplementary Fig. **10** shows the experimental and computational results of two-bit operation along with digital images of experimental tests. In Figs. **10**a and c, blue and green curves indicate stable and unstable paths of the two-bit system, respectively, based on the calculation of $\partial U/\partial\varphi_3 = 0$. The stability of the curve has been determined by the criterion whether the energy shows valley states (stable) or hilltop states (unstable). The red curves have been obtained from the experiments. The initial state is '00', in which $\varphi_1 = -62°, \varphi_3 = -31°$ (see the state (i) in Supplementary Fig. **10** a and b). We apply to the left-most polygon the gradual increase of φ_1 in the shape of the trapezoidal pulse input (Supplementary Fig. **9**c). Once the system departs from '00', it quickly jumps to '10' to avoid the unstable branch (see the change of states from (i) to (ii) in Supplementary Fig. **10**a and b). After that, it continues following the stable branch to reach '11' state as marked by state (iii) in Supplementary Fig. **10** a and b. See Supplementary Movie 6 for this sequential operation.

We also demonstrate '01' $\rightarrow$ '10'. Since the initial state is '00' from the previous test, we apply the pulse input (Supplementary Fig. **9**c) to φ_3 to obtain '01'. Thus, we reach the state (i) in Supplementary Fig. **10** c and d, in which $\varphi_1 = 0°, \varphi_3 = 28°$. Now we apply another pulse input (Supplementary Fig **9**d) to φ_1. It is noted that the initial state of the first bit shows $\varphi_1 = 0°$, and therefore, the pulse input starts from $0°$ and it is applied for $T/2$. Also, unlike

the monotonously increasing pulse input needed for the previous operation of '00' $\rightarrow$ '11', the operation of '01' $\rightarrow$ '10' requires the increasing – then decreasing – trend of the pulse input. This is due to the intermediary step of '11', which is positioned at the high value of φ_1. Therefore, for this operation, we use the trapezoidal waveform as shown in Supplementary Fig. **9**d. During the application of the pulse input, the state of the system changes from '01' (state (i) in Supplementary Fig. **10** c and d) to '11' (state (ii) in Supplementary Fig. **10** c and d). Then it becomes '10' (state (iii) in Supplementary Fig. **10** c and d).

Supplementary Note 10: Extension of TCO-based mechanical memory

To extend the TCO-based mechanical memory concept to multi-dimensions, we consider two different types of multiple bit systems. One is a two dimensional array of TCO-based memory units as shown in Supplementary Fig. **11**. Two adjacent memory units are connected by a linear spring. In this horizontal connection between two neighboring bits, planar arrangements of origami can enable tight network of unit cells through rotational motions. Supplementary Fig. **11**a shows that the application of clock-wise torque to the left bit changes the state of the right bit from '1' to '0'. On the other hand, if the two bits are connected in a diagonal direction and the same torque is applied to the left bit, the right bit transforms from '0' to '1'. Such origami cells arranged in a two-dimensional horizontal plane can interact with each other through rotational interactions. Note in passing here that a similar concept of plannar torsional network has been proposed for a wave dynamics application [2].

The other extended system is a multi-bit architecture based on controllable connections among cells (Supplementary Fig. **12**). That is, if we can have control over the connection of each cell (e.g., mechanical clutch that engages and disengages the mechanical connections among origami cells in a controllable way), we can possibly build a multi-bit system in combination of one- and two-bit memory cells. In Fig. **12**, we see an example of converting <000000> to

<101001> by arranging single- and double-bit operations in sequences, which are enabled by turning coupling on and off controllably. The concept of combining single and double bits for multi-bit operations has also been explored in [3].

In case we do not have a control over the inter-cell connections, it might be still possible to achieve multi-bit operations by employing origami cells with various potential levels (in a configuration analogous to the old rotary dial key of a safe). For example, if we consider a TCO memory system composed of graded n bits which are sorted according to energy barriers, we can operate bits one by one from the end of the system to the other end. This basic operation would require only direct coupling between neighboring cells, not any remote coupling mechanisms. By using this approach, we will be able to build a multi-bit system in a serial way without resorting to the inter-connection mechanism among neighboring cells. However, we expect that there would exist a practical limit in terms of the number of cells connected, because of the effect of friction.

While the aforementioned extensions of the origami cells in planar and serial fashions are conceptually feasible, we expect that the practical implementation of such architectures will be challenging, necessitating state-of-the-art design and fabrication techniques. For example, the multi-bit memory system illustrated in Supplementary Fig. **12** would require simple – yet robust and controllable – mechanical engagement mechanisms. Similarly, the planar system in Supplementary Fig. **11** will necessitate well-designed linkage structures for accurate torsional interactions among neighboring cells. If realized, however, the multi-dimensional architectures will be highly useful for various functionalities, e.g., mechanical memory storage and computing processes.

Supplementary References

[1] J. N. Reddy, Theory and analysis of elastic plates and shells (CRC press, Boca Raton, 2006).

[2] R. K. Pal, M. Schaeffer, and M. Ruzzene, Journal of Applied Physics **119**, 084305 (2016).

[3] E. G. Rieffel, W. H. Polak, Quantum computing: A gentle introduction (MIT press, Boston, 2011).

[4] T. Yamamoto, Y. A. Pashkin, O. Astafiev, Y. Nakamura, J. S. Tsai, Nature **425**, 6961: 941?944 (2003).
